# mRNA binding protein staufen 1-dependent regulation of pyramidal cell spine morphology via NMDA receptor-mediated synaptic plasticity

**DOI:** 10.1186/1756-6606-4-22

**Published:** 2011-06-02

**Authors:** Geneviève Lebeau, Luc DesGroseillers, Wayne Sossin, Jean-Claude Lacaille

**Affiliations:** 1 Department of Physiology, GRSNC, Université de Montréal, Montreal, Canada; 2 Department of Biochemistry, GRSNC, Université de Montréal, Montreal, Canada; 3 Department of Neurology and Neurosurgery, McGill University, Montreal, Canada

**Keywords:** Schaffer collateral synapses, RNA transport, late LTP, spontaneous activity-driven potentiation, spine morphogenesis

## Abstract

Staufens (Stau) are RNA-binding proteins involved in mRNA transport, localization, decay and translational control. The Staufen 1 (Stau1) isoform was recently identified as necessary for the protein synthesis-dependent late phase long-term potentiation (late-LTP) and for the maintenance of mature dendritic spines and synaptic activity in hippocampal CA1 pyramidal cells, strongly suggesting a role of mRNA regulation by Stau1 in these processes. However, the causal relationship between these impairments in synaptic function (spine shape and basal synaptic activity) and plasticity (late-LTP) remains unclear. Here, we determine that the effects of Stau1 knockdown on spine shape and size are mimicked by blocking NMDA receptors (or elevating extracellular Mg^2+^) and that Stau1 knockdown in the presence of NMDA receptor blockade (or high Mg^2+^) has no further effect on spine shape and size. Moreover, the effect of Stau1 knockdown on late-LTP cannot be explained by these effects, since when tested in normal medium, slice cultures that had been treated with high Mg^2+ ^(to impair NMDA receptor function) in combination with a control siRNA still exhibited late-LTP, while siRNA to Stau1 was still effective in blocking late-LTP. Our results indicate that Stau1 involvement in spine morphogenesis is dependent on ongoing NMDA receptor-mediated plasticity, but its effects on late-LTP are independent of these changes. These findings clarify the role of Stau1-dependent mRNA regulation in physiological and morphological changes underlying long-term synaptic plasticity in pyramidal cells.

## Introduction

Localization of mRNAs to synaptic sites and their subsequent translation have emerged as important mechanisms contributing to synapse-specific plasticity [1,2]. Thus, mRNA binding proteins (RBPs), which are key players in the transport of mRNAs, may be selectively implicated in various forms of plasticity that depend on the transport and local translation of specific transcripts. Staufen (Stau) [3,4], fragile × mental retardation protein (FMRP) [5,6], zipcode-binding proteins [7] and cytoplasmic polyadenyation element binding protein (CPEB) [8,9] are RBPs known to be implicated in mRNA dendritic localization and translation in neurons.

Notably, Stau is implicated in regulation of mRNAs required for memory formation in Drosophila and Aplysia [10,11]. In mammals, the two members of the Stau family, Stau1 and Stau2, are present in distinct ribonucleoprotein (RNP) complexes [12] and associate with different mRNAs [13]. Stau1 is required for the transport of mRNAs necessary for long-term potentiation at hippocampal synapses, as knockdown of Stau1 impaired dendritic transport of CaMKIIα mRNA in hippocampal neurons [3]. Moreover, downregulation of Stau1 also prevented the translation-dependent late phase LTP (late-LTP) induced by forskolin in CA1 pyramidal cells. However, the translation-independent early phase LTP was intact, suggesting an essential role of Stau1-dependent mRNA regulation in protein synthesis associated with late-LTP [14]. Interestingly, we recently found that Stau2-dependent regulation of mRNA was essential specifically for translation-dependent mGluR long-term depression, uncovering selective mechanisms of mRNA regulation for different forms of translation-dependent long-term synaptic plasticity [15].

Long-term changes in synaptic function are associated with changes in dendritic spines [16,17]. Indeed, we found that, in association with the impairment in late-LTP, Stau1 knockdown resulted in a shift from regular short spines to longer thin spines, suggesting a role in the formation and/or maintenance of mature spine shape [14]. However, since a form of NMDA-mediated plasticity, referred to as spontaneous activity-driven potentiation (SAP) [18], may be ongoing in our slice culture conditions and induce changes in spine shape [19-21], it is unknown whether the effects of Stau1 knockdown on late-LTP were due to its actions on spine morphogenesis, or vice versa. Thus, our aims were to test directly if preventing SAP by blocking NMDAR function (or elevating extracellular Mg^2+^) would influence the changes in dendritic spine morphology induced by Stau1 knockdown, and whether the changes induced by blocking SAP were in turn required for the effect on Stau1 knockdown on late-LTP. We found that while Stau1 is involved in spine morphogenesis through NMDAR-mediated SAP, the change in spine morphogenesis was not important for the effect of Stau1 on late-LTP.

## Methods

### Organotypic hippocampal slice cultures

All experiments were done in accordance with animal care guidelines at Université de Montréal, with the approval of the ethics committee at Université de Montréal (CDEA #10-003), and followed internationally recognized guidelines. Organotypic hippocampal slices were prepared and maintained in culture as previously described [14,22].

### siRNAs and transfections

siRNA target sequences for rat were as described [14]. Biolistic transfection of neurons in organotypic slice cultures was performed using a Helios gene gun (Bio-Rad, CA) following manufacturer's instructions as previously [14,22]. Electrophysiological recordings and cell imaging experiments were performed 48 hours after transfection and the experimenter was blind to transfection treatments.

### Electrophysiology

Individual slice cultures were transferred to a submerged-type recording chamber continuously perfused (at 1-2 ml/min) with artificial cerebrospinal fluid (ACSF) composed of (in mM): 124 NaCl, 2.5 KCl, 1.25 NaH_2_PO_4_, 1.3 MgSO_4_, 26 NaHCO_3_, 10 dextrose, 2.5 CaCl_2_, 2 μM adenosine, saturated with 95% O_2 _and 5% CO_2_, pH 7.4, as previously [14]. Field excitatory postsynaptic potentials (fEPSPs) were evoked by Schaffer collateral stimulation (30s^-1^) and recorded from CA1 stratum radiatum with a glass microelectrode (2-3 MΩ) filled with 2M NaCl, as previously [14].

### Imaging and morphological analysis

Slices were fixed with 4% paraformaldehyde and EYFP-transfected CA1 pyramidal neurons were randomly selected based on green fluorescence and characteristic morphology. Z-stacks were collected from the secondary branches of apical dendrites using a confocal laser scanning microscope LSM 510 (Carl Zeiss, Kirkland QC) and spines were analyzed using LSM 510 software as previously [14]. Briefly, spines were categorized in three different classes on the basis of length and shape [14]: 1 - filopodia, long protrusions (> 1 μm) without a spine head; 2 - elongated spines, long protrusions (> 1 μm) with a small head at the tip; and 3 - regular spines, short protrusions (< 1 μm) including stubby and mushroom-type spines.

## Results

**Figure 1:**
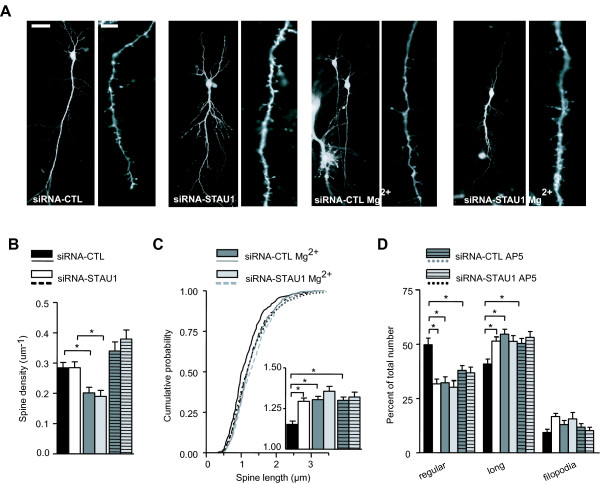
**Spine changes induced by Stau1 siRNA are prevented by NMDA receptor blockade and high Mg^2+^**. (A) Confocal images of representative YFP-expressing CA1 pyramidal cells (left) and apical dendrites (right) after co-transfection with siRNA-CTL or siRNA-STAU1 and maintained in normal or elevated Mg^2+ ^(12 mM) to impair NMDAR function. (B) Spine density was reduced by high Mg^2+ ^treatment but not with the NMDA receptor antagonist AP5 (100 μM), and was unchanged by Stau1 siRNA transfection. (C) Cumulative plots of the distribution of spine length for each condition, with summary bar graph of spine length in the inset, showing increased spine length in medium containing high Mg^2+ ^or AP5. Spine length was increased after Stau1 siRNA transfection in normal medium but not in Mg^2+ ^and AP5 treated slices. (D) Summary bar graph of number of regular, elongated and filopodia types of spines in each condition, showing decrease in regular and increase in elongated spines in high Mg^2+ ^or AP5. Stau1 siRNA transfection decreased regular and increased elongated spines in normal medium but not in high Mg^2+ ^or AP5, suggesting that Stau1 effects on spine shape are due to actions on endogenous NMDA receptor-mediated plasticity. Scale bars 25 μm, 5 μm. *, P < 0.05, t-test. Error bars represent s.e.m.

To examine the effect of Stau1 downregulation on dendritic spine morphology of CA1 pyramidal cells, organotypic hippocampal slice cultures were biolistically cotransfected with either siRNA-CTL or siRNA-STAU1 and plasmid coding for EYFP, as previously [14]. Confocal imaging of EYFP-labelled cells showed no apparent alteration in the general dendritic arborisation of transfected cells in any groups (Figure 1A). To prevent SAP, slice cultures were transfected and maintained for 48 h in medium containing elevated extracellular Mg^2+ ^concentration (12 mM) or the selective NMDA receptor antagonist AP5 (100 μM) [19-21]. In siRNA-CTL transfected cells, spine density was reduced in high Mg^2+ ^but not in AP5 (Table 1 and Figure 1B). The different effect may be due to additional actions of high Mg^2+^, such as inhibition of transmitter release [23], which might affect spine density. In siRNA-STAU1 transfected cells, spine density was unchanged relative to siRNA-CTL cells in any condition (Table 1), indicating no significant loss of spines after Stau1 knockdown, consistent with previous report [14].

**Table 1:** Spine changes induced by NMDA receptor blockade, high Mg^2+ ^and Stau1 siRNA treatment.

	siRNA-CTL			siRNA-STAU1		
	
	Normal medium	High Mg^2+ ^(12 mM)	AP5 (100 μM)	Normal medium	High Mg^2+ ^(12 mM)	AP5 (100 μM)
Spine density (spine/μm)	0.29 ± 0.02	0.21 ± 0.02*	0.34 ± 0.03	0.28 ± 0.02	0.19 ± 0.02	0.34 ± 0.03

Spine length (μm)	1.15 ± 0.02	1.3 ± 0.02*	1.3 ± 0.02*	1.29 ± 0.02^§^	1.35 ± 0.03	1.32 ± 0.03

Spine shape (% of total)						

Regular	49.75 ± 3.1	32.3 ± 2.7*	37.9 ± 2.2*	31.77 ± 2.2^§^	30.19 ± 3	36.8 ± 2.6

Elongated	40.92 ± 2.3	54.68 ± 2.3*	50.3 ± 2.3*	51.49 ± 2^§^	51.35 ± 2.7	53.1 ± 2.7

Filopodia	9.33 ± 1.5	13.02 ± 1.9	11.8 ± 1.6	16.74 ± 1.4	15.57 ± 3	10.2 ± 1.5

- Data expressed as mean ± s.e.m from 16-27 neurons per group, 4-8 independent experiments per group; in total 3129 protrusions were analyzed from 101 neurons.

- * indicates significant difference in siRNA-CTL relative to normal medium; P < 0.05, ANOVA.

- ^§ ^indicates significant difference in siRNA-STAU1 relative to siRNA-CTL in individual condition; P < 0.05, ANOVA.

Although spine density was not affected by Stau1 down-regulation, spine length and shape were modified (Table 1). Interestingly, blocking SAP had the same effect on spine length and shape as Stau1 knockdown. Indeed, spine length was increased in medium containing high Mg^2+ ^or AP5 (compared to normal medium) in siRNA-CTL transfected cells (Table 1 and Figure 1C), consistent with the idea that impairing NMDAR-mediated SAP prevents the formation of mature short spines. A similar increase in spine length was also observed in siRNA-STAU1 transfected cells (compared to siRNA-CTL) in normal medium as previously reported [14]. However, siRNA-STAU1 transfection in slices incubated in high Mg^2+ ^or AP5 had no further effect on spine length (compared to siRNA-CTL CTL in high Mg^2+ ^or AP5, respectively) (Table 1 and Figure 1C), suggesting that NMDAR-mediated SAP blockade occludes Stau1 knockdown consequences on spine length. Likewise in the case of spine shape, changes in the proportion of regular and elongated spines were similar in siRNA-CTL transfected cells treated with high Mg^2+ ^or AP5 (compared to normal medium) and in siRNA-STAU1 transfected cells in normal medium (compared to siRNA-CTL in normal medium): a decrease in regular spines and an increase in elongated spines (Table 1 and Figure 1D). Once again, there was no further effect of high Mg^2+ ^or AP5 medium in siRNA-STAU1 transfected cells (relative to siRNA-CTL in high Mg^2+ ^or AP5, respectively). These results indicate that NMDAR-mediated SAP blockade occludes Stau1 knockdown consequences on spine shape. Overall, these results suggest that SAP, mediated by NMDA receptors, leads to changes in spine size and shape over time in slice cultures and that these effects require Stau1. Thus, blocking either NMDA receptor mediated activity or Stau1 expression has the same effect on spine size and shape, and there are no additive effects when the two treatments are combined.

**Figure 2:**
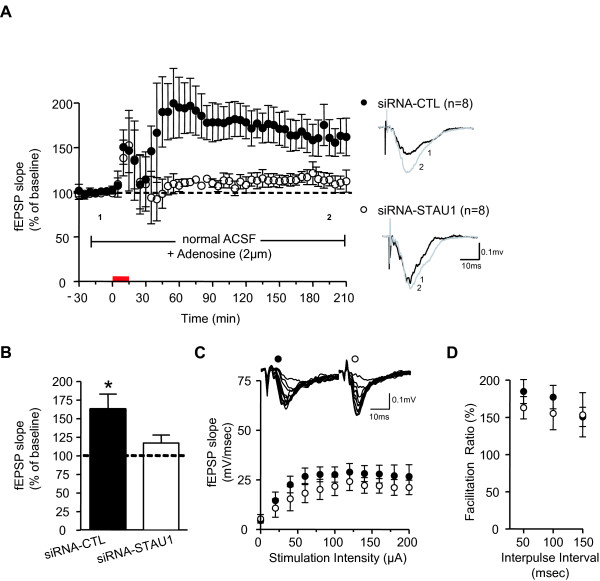
**Impairment of FSK-induced L-LTP after Stau1 knockdown during high Mg^2+ ^treatment**. (A) Potentiation of fEPSP slope induced by FSK application (50 μM, 15 min) in cultured slices maintained in medium containing high Mg^2+ ^for 48 hours after siRNA-CTL or siRNA-STAU1 transfection. For electrophysiological experiments, slices were tested in conditions with normal NMDA receptor function. Corresponding field potentials before (black line) and after (gray line) FSK application are shown at right. (B) Summary bar graph showing changes in fEPSPs slope 200 min post-FSK application. Significant L-LTP was present in slices transfected with siRNA-CTL but absent in slices transfected with siRNA-STAU1, indicating that Stau1 knockdown still prevents L-LTP after siRNA-STAU1 transfection in high Mg^2+^. *, P < 0.05, t-test. Error bars represent s.e.m. (C-D) Stau1 siRNA transfection did not affect basal synaptic transmission (C, input-output function; D, paired-pulse facilitation ratio).

Next, we determined if blocking the changes in spine morphology during SAP caused the loss of late-LTP seen with Stau1 down-regulation. Electrophysiological experiments were performed after maintaining slice cultures in elevated Mg^2+ ^medium for 48 hours after siRNA transfection. We took advantage of the fact that whereas biolistic DNA plasmid transfection in organotypic slice cultures lead to only a small percentage of transfected neurons (< 10%), delivery of siRNAs is much more efficient [14]. Using a fluorescently labelled siRNA (cyanine-3-tagged control siRNA) and confocal microscopy, high levels of siRNA are detected in most of the superficial principal neurons in slices, where the electrophysiological recordings are performed (see Figure 2 in [14]). The higher transfection efficiency may be due to the requirement for plasmid DNAs to penetrate not only the plasma membrane but also the nuclear membrane for effectiveness, while siRNA is effective in the cytoplasm. Extracellular field potentials (fEPSPs) were recorded in CA1 hippocampus in normal ACSF (containing normal extracellar Mg^2+^), and forskolin (FSK; 50 μM, 15 min) was used for chemical induction of late-LTP, as previously [14]. This form of L-LTP is NMDA receptor-mediated and is blocked by actinomicyn D [14]. Application of FSK induced a potentiation of fEPSPs lasting at least 3.5 h in slices transfected with siRNA-CTL (fEPSP slope 161.71% ± 20.18% of control; n = 8; P < 0.05) (Figure 2A and 2B). In slices transfected with siRNA-STAU1, FSK-induced late-LTP was blocked (116.09% ± 10.68% of control; n = 8; P > 0.05) (Figure 2A and 2B). The spontaneous synaptic activity which is induced by FSK application to activate NMDA receptors and which results in transient depression of fEPSPs [14] was similarly observed in both groups. Moreover, basal evoked synaptic transmission was unchanged after knockdown of Stau1, as shown by input-output function (n = 6 to 9; P > 0.05) (Figure 2C) and paired-pulse facilitation ratio (at intervals of 50 to 150 ms) of fEPSPs (n = 6 to 9; P > 0.05) (Figure 2D). Since under these conditions both siRNA-CTL and siRNA-STAU1 treated cultures had the same changes in spine shape and size due to the previous block of SAP, these changes cannot explain the loss of late-LTP in the Stau1 knockdown.

## Discussion

Our principal findings suggest that the mRNA binding protein Stau1 is implicated in the transport or regulation of mRNAs that are involved in long-term alterations of pyramidal cell dendritic spine morphology through NMDA receptor-mediated synaptic plasticity. NMDA receptors are crucial for synaptic plasticity and learning and memory [24]. During LTP induction, Ca^2+ ^entry through NMDAR activates multiple signalling pathways [25]. The maintenance of the enduring changes in synaptic efficacy consists in two phases. An early phase (early-LTP) is protein synthesis-independent and is characterized by phosphorylation of pre-existing proteins present at the synapse and synaptic insertion of AMPA receptors [26]. A longer-lasting late phase of LTP (late-LTP) is transcription- and translation-dependent [27] and is presumably associated with structural alterations of synapses that are reflected in part by changes in dendritic spine morphology [20]. Indeed, normal NMDA receptor function is thought to support morphological and structural stability of spines [28] and blockade of NMDA receptor activity favours the formation of immature type of spines [29]. In addition, spontaneous activity in hippocampal slice cultures induces NMDAR-mediated potentiation of synaptic transmission, referred to as spontaneous activity-driven potentiation (SAP; [18]). During SAP, NMDAR-activity leads to synaptic insertion of GluR1 [18] and a stable increase in spine size [19], analogous to changes occurring during LTP [21]. Our findings are consistent with a model in which NMDAR-dependent signalling activates Stau1-dependent mechanisms of mRNA regulation during LTP and SAP induction, which lead to translation of mRNAs necessary for a long-lasting increase in synaptic efficacy, ultimately reflected as stable increases in mature spine shape. Stau1 effects on spine morphology and late LTP may also reflect different cell biological processes (both requiring NMDA receptors and/or transmitter release from presynaptic neurons), with spine morphology changes reflecting a slow function over a much longer time scale (dependent on SAP and RNA transport), and late LTP implicating a more rapid regulation of RNA transport. In addition, we cannot rule out the possibility that more subtle effects of Stau1 on spine morphology, undetected in the present study, may be related to its blocking effect on late LTP.

Our findings that knockdown of Stau1 impairs late-LTP, without affecting the early form of LTP or basal transmission [14], is consistent with Stau1 regulation of the translation/transport of mRNAs, but the specific role that Stau1 plays is still not clear. In the context of late-LTP, the mRNAs that are translated can consist of previously transcribed plasticity-related mRNAs that were transported constitutively to synapses prior to LTP induction [27] or newly transcribed mRNAs that need to be transported to the activated synapse for local translation [30]. Activity-dependent localization of specific mRNAs in dendrites has been demonstrated in cultured neurons [31-33] and *in vivo *[34,35], providing compelling support for the idea that glutamate receptor signalling may regulate dendritic mRNA transport and docking at postsynaptic sites in long-term plasticity. Stau1 was shown to be involved in the constitutive transport in dendrites of plasticity-related mRNAs such as CaMKIIα mRNA [3] supporting a role for Stau1 in constitutive transport of plasticity-related mRNAs. Mutant mice with impaired dendritic translation of CaMKIIα mRNA show impairments in late-LTP and hippocampal-dependent memory [36]. Thus, CaMKIIα mRNA is a likely mRNA regulated by Stau1 during both LTP [36] and SAP [18]. It remains to be determined if other mRNAs known to be regulated in late-LTP, like Arc and PKMζ [37-39], are similarly regulated in Stau1-dependent fashion. Thus, LTP and SAP could be blocked due to the lack of these mRNAs in dendrites when plasticity is induced. It is also possible that Stau1 is critical for the translation/transport of mRNAs induced by LTP. Indeed, neuronal activity induced by depolarization was shown to significantly increase RNP containing Stau2 in dendrites of cultured neurons [40], indicating a role of Stau2 in activity-dependent transport of mRNA. Further studies will be required to define the precise manner by which Stau1 regulates SAP and LTP.

In a recent study with a mutant mouse expressing a truncated Stau1 protein lacking the functional RNA-binding domain 3 (RBD3), cultured hippocampal neurons displayed deficits in dendritic delivery of Stau1-containing RNP, as well as reduced dendritic tree and fewer synapses, indicating that Stau1 is crucial for synapse development *in vitro *[41]. These mice showed impaired locomotor activity but no significant deficit in hippocampal-dependent learning and memory, although the lack of a deficit in hippocampal function may reflect compensatory changes involving other proteins or genetic background effects [41]. It would be interesting to determine if impairments in late-LTP are present in these mice to examine if Stau1-dependent mRNA regulation in long-term plasticity is dependent on the functional RBD3 domain.

In conclusion, we found that Stau1 involvement in spine morphogenesis is dependent on NMDA receptor-mediated plasticity in hippocampal pyramidal cells. We also found that Stau1 is required for late-LTP, independently of its role in spine morphogenesis. These findings clarify the role of Stau1-dependent mRNA regulation in the physiological and morphological changes at pyramidal cell synapses during long-term plasticity underlying hippocampal-dependent learning and memory.

## Competing interests

The authors declare that they have no competing interests.

## Authors' contributions

This study was conceived and designed by JCL, WS and LD. Experiments were conducted by GL. The manuscript was written by GL, JCL, WS and LD. The entire project was supervised by JCL. All authors read and approved the final manuscript

## Acknowledgements

This research was supported by the Canadian Institutes of Health Research (CIHR Team in Memory Grant, grant #CTP-79858, JCL; CIHR grant MOP 15121, WS), Fonds de la recherche en santé du Québec (Groupe de recherche sur le système nerveux central; grant #5249; JCL, WS, LD) and the Canada Research Chair Program (Canada Research Chair in Cellular and Molecular Neurophysiology; grant #950-213424; JCL). GL was supported by a Savoy Foundation studentship. The authors would like to thank Julie Pepin and Catherine Bourgeois for excellent technical assistance.
